# Cholesterol Contributes to Diabetic Nephropathy through SCAP-SREBP-2 Pathway

**DOI:** 10.1155/2013/592576

**Published:** 2013-11-27

**Authors:** Hong Sun, Yang Yuan, Zi-Lin Sun

**Affiliations:** Department of Endocrinology, Zhongda Hospital, Institute of Diabetes, Medical School, Southeast University, Nanjing 210009, China

## Abstract

Diabetic nephropathy (DN) has been associated with the presence of lipid deposition. We hypothesized that the disruption of intracellular cholesterol feedback may contribute to DN. Diabetes was induced by high fat/sucrose diet and low-dose intraperitoneal injection of streptozocin (STZ) in male Sprague-Dawley rats. Then diabetic rats were randomly divided into two groups: untreated diabetic group (DM) and atorvastatin-treated group (DM + AT). We found that the levels of serum blood urea nitrogen and creatinine, as well as 24-hour urine protein and urinary neutrophil gelatinase-associated lipocalin, were significantly increased in diabetic rats. This result indicated that the diabetic rats suffered from functional renal damage. We also observed lipid droplet accumulation and increase in 3-hydroxy-3-methylglutaryl coenzyme A reductase (HMG-CoAR), low density lipoprotein receptor (LDLr), sterol regulatory element binding protein-2 (SREBP-2), and SREBP-cleavage activating protein (SCAP) in the kidneys of diabetic rats. However, atorvastatin ameliorated renal lipid accumulation and improved the renal function of diabetic rats despite an increase in mRNA and protein expressions of HMG-CoAR, LDLr, and SREBP-2. These results demonstrated that intracellular cholesterol feedback regulation is disrupted in rats with type 2 diabetes, thereby causing renal cholesterol accumulation. Atorvastatin ameliorated renal cholesterol accumulation by reducing renal cholesterol synthesis.

## 1. Introduction

Type 2 diabetes mellitus (T2DM) results from a progressive insulin secretion defect on the background of insulin resistance, leading to the loss of glycemic control and eventual diabetes complications, such as diabetic nephropathy (DN). DN is a major cause of end-stage renal disease. Evidence has suggested that dyslipidemia and renal accumulation of lipids have a pathogenetic function in the development and progression of DN [1–5]. Most studies have focused on the dysregulation of triglycerides in the kidneys. However, the mechanism by which cholesterol contributes to DN progression remains unknown.

Increasing cholesterol uptake and synthesis can result in lipid deposition in the kidneys, thus causing renal dysfunction. Low density lipoprotein receptor (LDLr) and 3-hydroxy-3-methylglutaryl coenzyme A reductase (HMG-CoAR), which serve important functions in maintaining cholesterol uptake and synthesis, respectively, are predominantly regulated by SREBP-2 in the human mesangial cell line [6, 7]. SREBP-cleavage activating protein (SCAP) is the chaperone of SREBP-2. When cells demand cholesterol, SCAP shuttles SREBP-2 from the endoplasmic reticulum (ER) to the Golgi apparatus for activation by proteolytic cleavage [8]. The cleaved N-terminal fragments of SREBP-2 (nSREBP-2) are then translocated to the nucleus, where they activate LDLr and HMG-CoAR, resulting in increases in cholesterol uptake and synthesis. By contrast, the SCAP-SREBP complex is retained in the ER when cells contain sufficient cholesterol, thereby downregulating LDLr and HMG-CoAR expression. This feedback regulation mediated by SCAP can prevent the overloading of intracellular cholesterol under physiological conditions [9, 10].

Atorvastatin was first introduced to clinical practice as a lipid-lowering agent and was thereafter found to have antioxidative and anti-inflammatory effects. Numerous clinical trials have consistently demonstrated the beneficial effects of atorvastatin on the prevention of cardiovascular disease and the improvement of outcomes in diabetic patients [11–14]. Several studies have shown that atorvastatin can improve estimated glomerular filtration rate, decrease serum creatinine (Cr), and exhibit beneficial effects on the renal function of diabetic patients [15, 16]. However, the mechanism remains unknown.

This study aimed to investigate whether or not SCAP-mediated intracellular cholesterol feedback is disturbed in the kidneys of rats with type 2 diabetes induced by high-fat/sucrose diet and low-dose streptozocin (STZ). We also investigated the function of atorvastatin, an HMG-CoAR inhibitor, in the renal lipid metabolism of type 2 diabetic rats.

## 2. Materials and Methods

### 2.1. Animal Model

Male Sprague-Dawley rats weighing 150 g to 170 g were purchased from Shanghai Sipprbk Laboratory Animals Ltd. (Shanghai, China). The rats were housed in polypropylene cages and maintained under controlled room temperature (22 ± 2°C) and humidity (60 ± 5%) with 12 h : 12 h light : dark cycle. Housing and animal experiments were approved by the Jiang Su Animal Care and Use Committee according to institutional guidelines and national animal welfare. After one week of adaptation, the rats were randomized into two groups. The control rats (NC) were fed with regular food, whereas the other group was fed with a high fat/sucrose diet (67% standard chaw, 10% lard, 20% sugar, 2.5% cholesterol, and 0.5% sodium cholate) to induce diabetes [17]. Four weeks later, the rats on the high fat/sucrose diet were injected with 30 mg/kg STZ (dissolved in citrate buffer, pH 4.5) intraperitoneally, whereas the NC were injected with the same volume of citrate buffer. Only the rats with a nonfasting blood glucose of ≥16.7 mmol/l were considered diabetic and selected for further studies [18]. Two weeks after diabetes was induced, the diabetic rats were randomly divided into two groups: untreated diabetic group (DM) and atorvastatin-treated group (DM + AT, administered extract at 10 mg/kg body weight/day) [19]. The 24-hour urine of rats was collected in individual metabolic cages to measure 24-hour urine protein and urinary neutrophil gelatinase-associated lipocalin (u-NGAL) levels. The rats were then weighed and sacrificed. Blood was collected to test Cr, blood urea nitrogen (BUN), total triglycerides (TG), total cholesterol (TC), and low density lipoprotein (LDL). The kidneys were perfused with saline solution and then removed and weighed. Part of the kidneys was fixed in 10% neutral formalin and embedded in paraffin for hematoxylin-eosin (HE) staining, periodic acid Schiff (PAS) staining, and periodic acid-silver methenamine (PASM) staining. Part of the kidneys was fixed in 4% paraformaldehyde and then dehydrated and embedded in OCT for Oil Red O staining. The left tissue was immediately stored at −80°C for real-time reverse transcription polymerase chain reaction (RT-PCR) and western blot.

### 2.2. Biochemical Assay

Serum Cr, BUN, TG, TC, and LDL were determined using enzymatic kits (Kanto Chemical Co., Inc., Tokyo, Japan). The 24-hour urine protein was measured using a Coomassie brilliant blue protein assay (Jiancheng Bioengineering Institute, Nanjing, Jiangsu, China). u-NGAL was measured using the ELISA method by Uscn (Wuhan, China).

### 2.3. Observation of Lipid Accumulation

The renal lipid accumulation was evaluated by Oil Red O staining. Briefly, samples were fixed with 4% paraformaldehyde and then stained with Oil Red O for 30 min. Finally, samples were counterstained with hematoxylin for 5 min. Results were examined by light microscopy.

### 2.4. Renal Histology

Sequential paraffin-embedded tissue sections from the renal cortex were cut. Cross sections (3 *μ*m) were placed on gelatin-coated slides and used for HE staining, PAS, and PASM staining.

### 2.5. Real-Time RT-PCR

Total RNAs were isolated from cells or kidney homogenates from type 2 diabetic rats using TRIzol reagent (Invitrogen, USA) and reverse transcripted to complementary DNA using Promega RT kit (Promega, Madison, WI, USA). Equal amounts of the product of the reverse transcription reaction were subjected to PCR amplification. The mRNA levels of HMG-CoAR, LDLr, SREBP-2, and SCAP were normalized with the GAPDH mRNA level. PCR primers were synthesized by GenScript Co. Ltd. (Nanjing, China). The primer sequences and amplified products lengths are shown in Table 1.

### 2.6. Western Blot

Protein was separated on 7.5% SDS-PAGE gel. Polyvinylidene fluoride membrane (Millipore Corporation, Bedford, MA, USA) was used for transfer and then blocked for 1 h at room temperature with 5% bovine serum albumin in Tris-buffered saline containing 0.05% Tween 20 (TBST). Subsequently, blots were washed and incubated overnight at 4°C in TBST containing 1% bovine serum albumin with a 1 : 500 dilution of HMG-CoAR, LDLr, SREBP-2, and SCAP antibody as well as *β*-actin antibody. The rabbit anti-SREBP-2 antibody can detect both the precursor segment and mature segment of SREBP-2 protein. Membranes were washed thrice with TBST, incubated with a secondary antibody (1 : 5000 dilutions in TBST containing 1% bovine serum albumin; Santa Cruz Biotechnology) for 120 min at room temperature, and then washed thrice with TBST. After the chemiluminescence reaction (Pierce, Rockford, IL, USA), bands were detected by exposing blots to X-ray films for the appropriate time period. For quantitative analysis, bands were detected and evaluated for their densities by LabWorks software (UVP Laboratory Products, Upland, CA, USA), normalized for *β*-actin density.

### 2.7. Statistical Analysis

In all experiments, data were expressed as means with standard deviations and analyzed by SPSS 18.0 for Windows. Means of every two different groups were detected using the Student's *t*-test. *P* < 0.05 was considered statistically significant.

## 3. Result

### 3.1. Biochemical and Histological Characteristics of Type 2 Diabetic Rat Model

**Figure 1:**
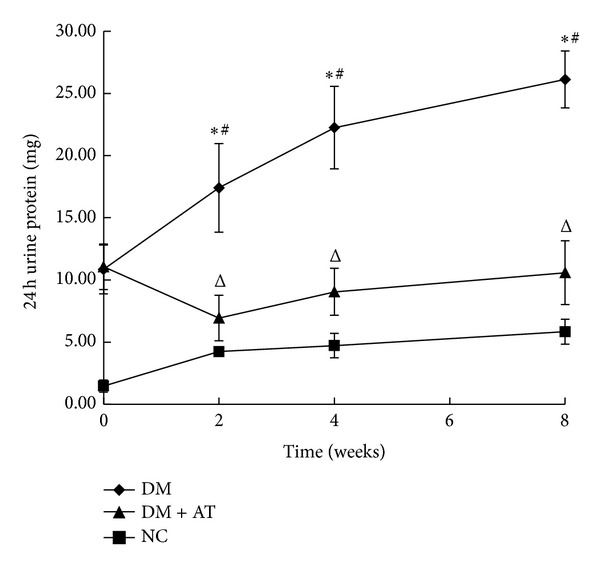
24-hour urine protein. A significant increase in 24-hour urine protein as observed in type 2 diabetic rats compared with the control rats; the amount of this protein continued to increase with the progress of diabetes. The 24-hour urine protein was improved after atorvastatin treatment for two weeks. Data are means ± SD. *P < 0.05 versus NC; ^#^ *P* < 0.05 2-week treatment versus 0-week treatment, 4-week treatment versus 2-week treatment, and 8-week treatment versus 4-week treatment; ∆, *P* < 0.05 versus DM.

**Figure 2:**
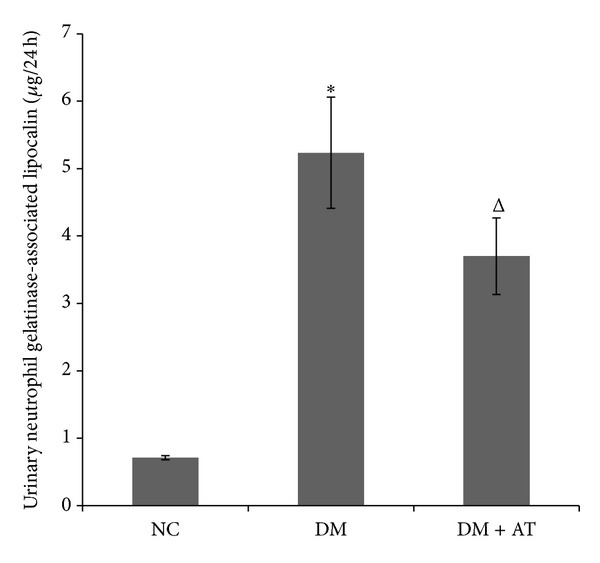
Urinary neutrophil gelatinase-associated lipocalin (u-NGAL) level. u-NGAL was significantly increased in diabetic rats but was reduced by atorvastatin treatment. Data are presented as means ± SD. *P < 0.05 versus NC; ∆, *P* < 0.05 versus DM.

Blood glucose was significantly increased after STZ injection followed by clear manifestations of diabetes, including polydipsia, diuresis, polyphagia, and weight loss. The levels of serum Cr, BUN, TG, TC, and LDL were markedly higher in untreated diabetic rats than in NC (Table 2). The 24-hour urine protein of the DM group was significantly increased compared with NC, and it continued to elevate with the progress of diabetes (Figure 1). u-NGAL was also elevated in the DM rats (Figure 2).

### 3.2. Lipid Accumulation in Kidney

**Figure 3:**
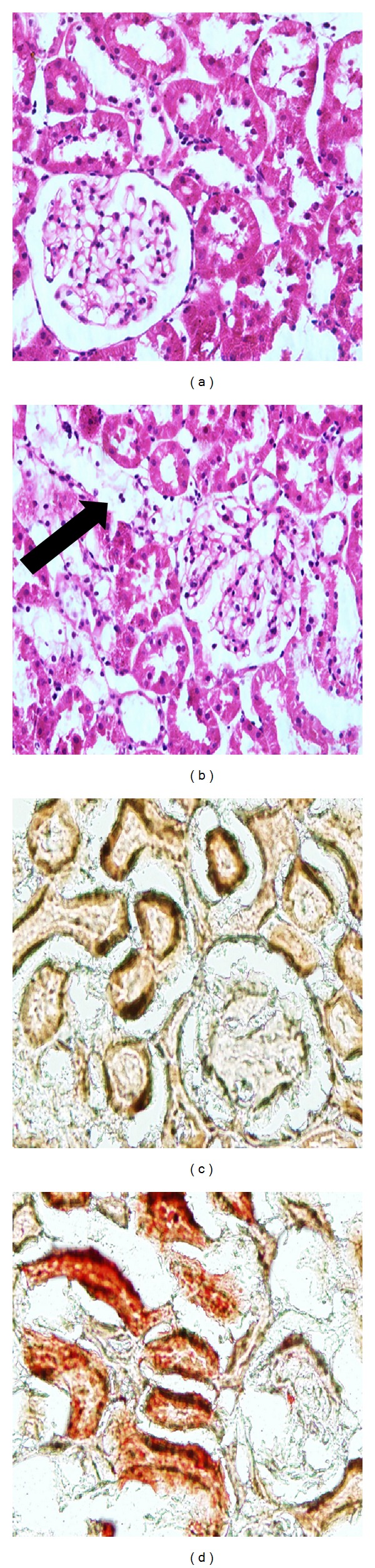
Renal HE (400×, (a) and (b)) and Oil Red O staining (200×, (c) and (d)). NC, (a) and (c); DM, (b) and (d). HE staining showed many vacuolar cells (black arrow) in the kidneys of the diabetic rats. Representative photomicrograph of the Oil-Red-O-stained renal tissues. Lipid droplets appear as red spots. Lipid droplets accumulated in the kidneys of the diabetic rats.

HE and Oil Red O (Figure 3) staining showed lipid droplet accumulation in the kidneys of the diabetic rats. However, no lipid was found in the kidneys of NC.

### 3.3. Expression of HMG-CoAR, LDLr, SREBP-2, nSREBP-2, and SCAP and in the Renal Cortex of Type 2 Diabetic Rat Model

**Figure 4:**
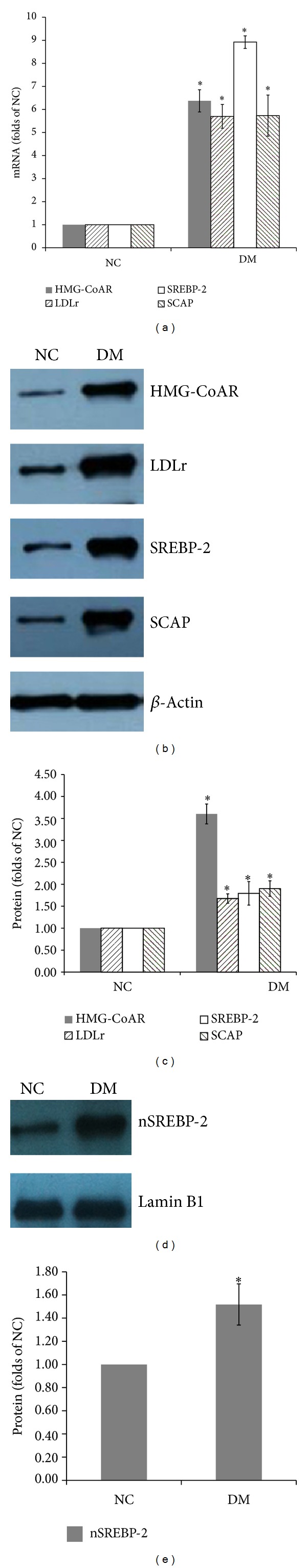
mRNA and protein expressions of HMG-CoAR, LDLr, SREBP-2, SCAP, and nSREBP-2. mRNA levels were determined by real-time RT-PCR as described in Section 2. GAPDH was used as a reference gene. Data are presented as means ± SD (a). The protein levels were examined by western blot (b). The histogram represents means ± SD of the densitometric scans of HMG-CoAR, LDLr, SREBP-2, and SCAP proteins, normalized by comparing with actin. This result is expressed as a percentage of control (c). The protein level and histogram of nSREBP-2. Data are means ± SD ((d) and (e)). *P < 0.05 versus NC.

The mRNA and protein expression of HMG-CoAR, LDLr, SREBP-2, nSREBP-2, and SCAP were significantly increased in type 2 diabetic rats compared with NC (Figure 4). These results suggest that the renal lipid accumulation of type 2 diabetic rats may be caused by the activation of the SCAP-SREBP pathway, which increases LDLr-mediated cholesterol uptake and HMG-CoAR-mediated cholesterol synthesis.

### 3.4. Effects of Atorvastatin on Metabolic Parameters

Atorvastatin treatment had no effect on the level of serum glucose and on increased KW/WT. The levels of serum TC and LDL were markedly reduced by the atorvastatin treatment. However, the decrease in TG was not significant (Table 2).

### 3.5. Effects of Atorvastatin on Renal Function

The Cr, BUN, 24-hour urine protein, and u-NGAL in the DM group were significantly increased compared with the NC group, indicating damaged renal function in these rats. By contrast, Cr, 24-hour urine protein, and u-NGAL were improved in the DM + AT group, but BUN had minimal change despite atorvastatin treatment (Table 2 and Figures 1 and 2).

### 3.6. Atorvastatin Inhibited Renal Lipid Accumulation and Ameliorated Renal Morphology

**Figure 5:**
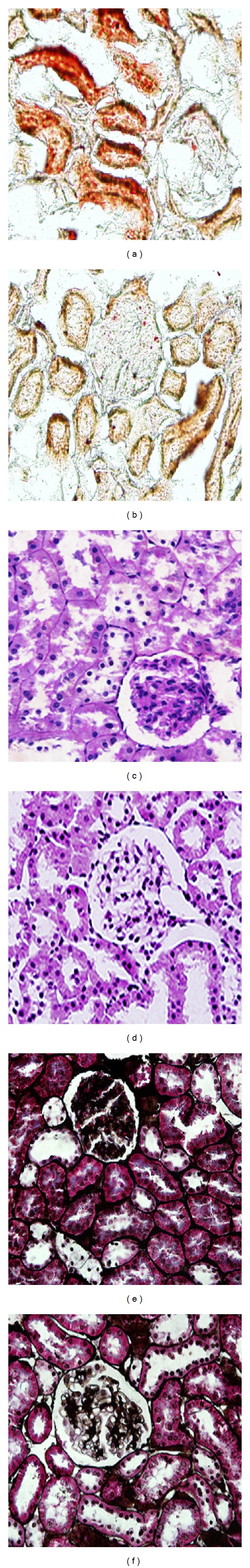
Oil Red O staining (200×, (a) and (b)). Increased Oil Red O staining was observed in the glomerular and tubular cells of diabetic rats, and treatment with atorvastatin 10 mg/kg/day reverses these changes. PAS staining (400×, (c) and (d)). Significant mesangial expansion was observed in the glomeruli of diabetic rats, and atorvastatin alleviated this change. (c) PASM staining (400×, (e) and (f)). PASM staining showed the basement membrane thickness in the glomeruli and tubules of diabetic rats, which were changed by atorvastatin treatment. NC, (a), (c), and (e); DM, (b), (d), and (f).

The significant increase in renal lipid droplets in the high fat/sucrose diet and low-dose STZ-induced type 2 diabetic rats was reversed by atorvastatin treatment. PAS staining showed mesangial expansion in the renal glomeruli. PASM staining showed the basement membrane thickness in the glomeruli and tubules of diabetic rats. Both were alleviated by atorvastatin (Figure 5).

### 3.7. Effects of Atorvastatin on Expression of HMG-CoAR, LDLr, SREBP-2, nSREBP-2, and SCAP in the Renal Cortex of Type 2 Diabetic Rats

**Figure 6:**
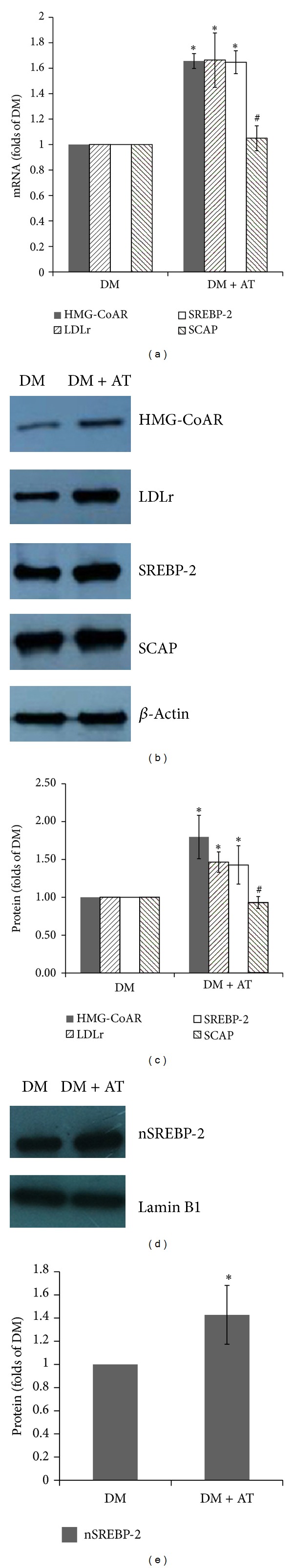
Effects of atorvastatin on mRNA and protein expression of HMG-CoAR, LDLr, SREBP-2, SCAP, and nSREBP-2. mRNA levels were determined by real-time RT-PCR as described in Section 2. GAPDH was used as a reference gene. Data are means ± SD (a). Protein levels were examined by western blotting (b). Histogram represents means ± SD of the densitometric scans of HMG-CoAR, LDLr, SREBP-2, and SCAP proteins, normalized by comparing with actin and expressed as a percentage of control (c). Protein level and histogram of nSREBP-2. Data are means ± SD ((d) and (e)). Atorvastatin treatment increased the mRNA and protein expression of HMG-CoAR, LDLr, SREBP-2, and nSREBP-2, but no change was observed in SCAP. *P < 0.05 versus DM; ^#^ *P* > 0.05 versus DM.

The mRNA and protein expression of HMG-CoAR, LDLr, SREBP-2, nSREBP-2, and SCAP were significantly increased in type 2 diabetic rats. Atorvastatin treatment increased the expression of HMG-CoAR, LDLr, SREBP-2, and nSREBP-2. However, the mRNA and protein expression of SCAP were unchanged by atorvastatin treatment (Figure 6).

## 4. Discussion

Appropriate animal models are important tools to reveal the underlying mechanisms of DN. In this study, the effects of atorvastatin on DN were assessed using a high fat/sucrose diet and STZ-induced diabetic rat models, which could closely mimic the clinical situation in humans. Atorvastatin acts as an HMG-CoAR inhibitor and has been extensively investigated in terms of its tolerability and safety, lipid-lowering effects, and capability to inhibit the development of diabetic renal diseases [15].

The most important findings of this study are as follows. (1) SCAP-mediated intracellular cholesterol feedback regulation was disrupted in the kidneys of high fat/sucrose diet and low-dose STZ-induced type 2 diabetic rats. HE and Oil Red O staining showed that lipid droplets accumulated in the glomeruli and tubular cells of the kidneys of diabetic rats. Furthermore, HMG-CoAR, LDLr, SREBP-2, nSREBP-2, and SCAP are upregulated in the kidneys of diabetic rats. Previous reports showed that increased SCAP expression and SCAP translocation from the ER to the Golgi apparatus cause the overexpression of nSREBP-2, thus upregulating HMG-CoAR and LDLr expressions [7]. Therefore, increasing HMG-CoAR-mediated cholesterol synthesis and LDLr-mediated cholesterol uptake possibly resulted in renal cholesterol accumulation in diabetic rats. (2) As an HMG-CoAR inhibitor, atorvastatin could alleviate dyslipidemia and renal lipid accumulation. Atorvastatin could also improve the renal morphology and function of diabetic rats and possesses a potential renoprotective role in the prevention of diabetic renal injury. In our study, the levels of serum TC and LDL were markedly reduced by atorvastatin treatment. Atorvastatin also improved renal function by reducing serum Cr level and 24-hour urine protein. Considering that the significant Oil Red O staining in the tubules and tubular dysfunction is an important component of diabetic renal disease, we also measured u-NGAL to evaluate the tubular function. NGAL, a small 25 kD protein, belonging to the “lipocalins” superfamily, is hyperproduced when renal tubules are injured. NGAL is a novel and promising tubular biomarker in the diagnostic field of diabetic renal disease [20, 21]. In our study, atorvastatin decreased u-NGAL in diabetic rats. All of these observations indicate that atorvastatin improves the function of diabetic renal glomeruli and tubules.

Accumulated evidence indicates that dyslipidemia serves an important function in the progression of kidney disease in patients with diabetes [22, 23]. In fact, lipid-lowering therapy by statins has been successful in the amelioration of renal function in patients with diabetic nephropathy [15, 16, 24, 25]. However, some animal studies show that statin treatment significantly improves renal function without affecting the plasma lipid profile [26–28]. Therefore, the relationship between dyslipidemia and renal function in diabetic patients remains unclear. Nevertheless, the finding that treatment with atorvastatin reversed the increased Oil Red O staining in the glomerular and tubular cells of diabetic rats is significant. The upregulation of HMG-CoAR, LDLr, SREBP-2, and nSREBP-2 was further promoted; this result is consistent with the effects of intestinal treatment [29]. However, the mRNA and protein expression of SCAP were unchanged; we hypothesized that the intracellular SCAP was sufficient to meet the need for the increasing transfer of SREBP-2. Atorvastatin inhibited the activity of HMG-CoAR but increased SREBP-2-mediated HMG-CoAR and LDLr expressions, suggesting a potential mechanism that the downregulation of cholesterol synthesis by atorvastatin could cause a compensatory increase in cholesterol absorption. Considering that Oil Red O staining did not exhibit lipid deposition after atorvastatin treatment, we hypothesized that the main reason for renal lipid deposition in diabetic rats is HMG-CoAR-mediated cholesterol synthesis instead of LDLr-mediated cholesterol uptake. This increasing HMG-CoAR-mediated cholesterol synthesis was also considered as the reason for acute renal injury, in which cholesterol accumulation appears as a ubiquitous response [30]. Statin treatment also prevents cholesterol accumulation in cultured HK-2 cells following Fe-mediated injury and further supports this link [31]. Furthermore, we conducted PAS and PASM staining to determine the subtle structural changes in the renal glomeruli and tubules. The result showed mesangial expansion in the renal glomeruli and basement membrane thickness in the glomeruli and tubules of diabetic rats, but atorvastatin alleviated these changes. The presence of lipids in renal cells upregulates intracellular signaling pathways involved in inflammatory and fibrogenic responses, both of which are components of progressive renal injury [32]. Based on our results, we hypothesized that the improvement of the renal morphology and function after atorvastatin treatment may be mediated by decreasing cholesterol synthesis in the kidneys of diabetic rats.

In conclusion, the activation of the SCAP-SREBP-2-HMG-CoAR/LDLr pathway caused renal cholesterol accumulation in high fat/sucrose-fed and STZ-induced rat models, resulting in diabetic renal injury. Nevertheless, atorvastatin could reduce cholesterol synthesis in the kidneys, thereby improving renal morphology and function in diabetic rat models. Atorvastatin is a potential novel approach to treat diabetic renal damage.

## Figures and Tables

**Table 1:** The primers for real-time RT-PCR.

Genes	Primers
HMG-CoAR	5′-TGTTGCCATCAACGACCCCTT-3′ sense
	5′-CTCCACGACATACTCAGCA-3′ antisense

SREBP-2	5′-CACTCACGCTCCTCGGTCAC-3′ sense 5′-GGATAAGCAGGTCTGTAGGTTGG-3′ antisense

SCAP	5′-GCCAGAGTGGTATGTGGGTGC-3′ sense
	5′-CCAGTTGGAATGCTCGGGAC-3′ antisense

GAPDH	5′-TGTTGCCATCAACGACCCCTT-3′ sense 5′-CTCCACGACATACTCAGCA-3′ antisense

HMG-CoAR: 3-hydroxy-3-methylglutaryl coenzyme A reductase; SCAP: sterol regulatory element binding protein (SREBP) cleavage-activating protein.

**Table 2:** Biochemical parameters of NC, DM, and DM + AT groups at the end of intervention study (*n* = 5).

Group	NC	DM	DM + AT
WT (g)	460.40 ± 21.41	303.00 ± 18.57*	296.00 ± 49.66
KW/WT	0.008 ± 0.000	0.014 ± 0.002*	0.014 ± 0.001
FBG (mmol/L)	6.40 ± 0.61	25.52 ± 2.87*	23.48 ± 5.97
Cr (umol/L)	37.00 ± 3.46	67.00 ± 14.35*	35.40 ± 5.03^Δ^
BUN (mmol/L)	5.32 ± 0.28	9.26 ± 1.44*	10.94 ± 2.57
TG (mmol/L)	0.38 ± 0.09	1.89 ± 0.25*	1.08 ± 0.88
TC (mmol/L)	1.70 ± 0.24	11.60 ± 3.98*	4.43 ± 2.35^Δ^
LDL (mmol/L)	0.76 ± 0.07	4.72 ± 0.90*	1.87 ± 0.73^Δ^

Data are presented as means ± SD. *P < 0.05 versus NC; ^Δ^ *P* < 0.05 versus DM.
